# Peritoneal Dialysis in an Extremely Low Birth Weight Neonate with Ileostomy

**DOI:** 10.5005/jp-journals-10071-23167

**Published:** 2019-05

**Authors:** Amita Kaul, Kapil Jadhav, Sachin Shah

**Affiliations:** 1-3 Department of Pediatrics, Surya Mother and Child Superspecialty Hospital, Pune, Maharashtra, India

**Keywords:** Acute peritoneal dialysis, Extremely low birth weight, Ileostomy

## Abstract

**Background:**

Acute kidney injury (AKI) is a common complication in sick, extremely low birth weight (ELBW) neonates. Peritoneal dialysis (PD) is the treatment modality but is seldom attempted in this patient population. We present a 40-day-old ELBW neonate previously operated for necrotizing enterocolitis with ileostomy who developed AKI. Peritoneal dialysis was started by a modifying intercostal drain.

**Results:**

Doing a simple procedure and using a few modifications helped the baby to come out of acute kidney injury.

**Conclusion:**

Peritoneal dialysis can be technically quite challenging in ELBW neonates but is possible with certain innovative modifications.

**Key message:**

Challenging technical problems in potentially fatal conditions sometimes respond to simple innovation. Initiation of early peritoneal dialysis in a sick ELBW with ileostomy and AKI helped saved her life.

**How to cite this article:**

Kaul A, Jadhav K, Shah S. Peritoneal Dialysis in an Extremely Low Birth Weight Neonate with Ileostomy. Indian J Crit Care Med 2019;23(5):232–233.

## BACKGROUND

Peritoneal dialysis (PD) for acute kidney injury (AKI) of newborns has been performed safely and regularly. Acute kidney injury is a known phenomenon in extremely low birth weight (ELBW) neonates. Doing PD in ELBW neonates requires expertise. Probable delay in diagnosis and initiation of treatment makes prognosis poor for ELBW neonates with AKI. We report an ELBW with ileostomy who developed AKI due to fungal sepsis on whom we performed peritoneal dialysis using an innovative modification of the catheter.

## CASE REPORT

**Fig. 1:**
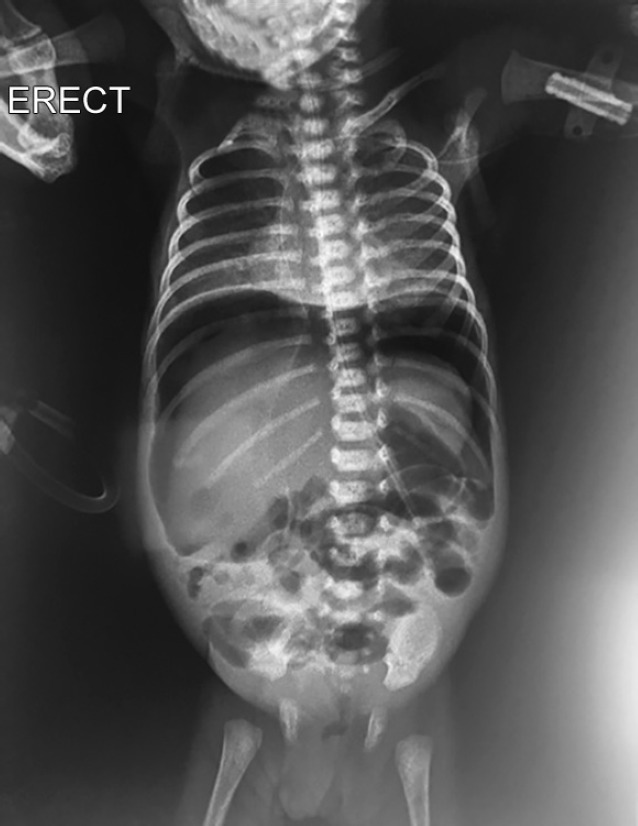
X-ray picture of pneumoperitoneum after which ileostomy was done

**Fig. 2:**
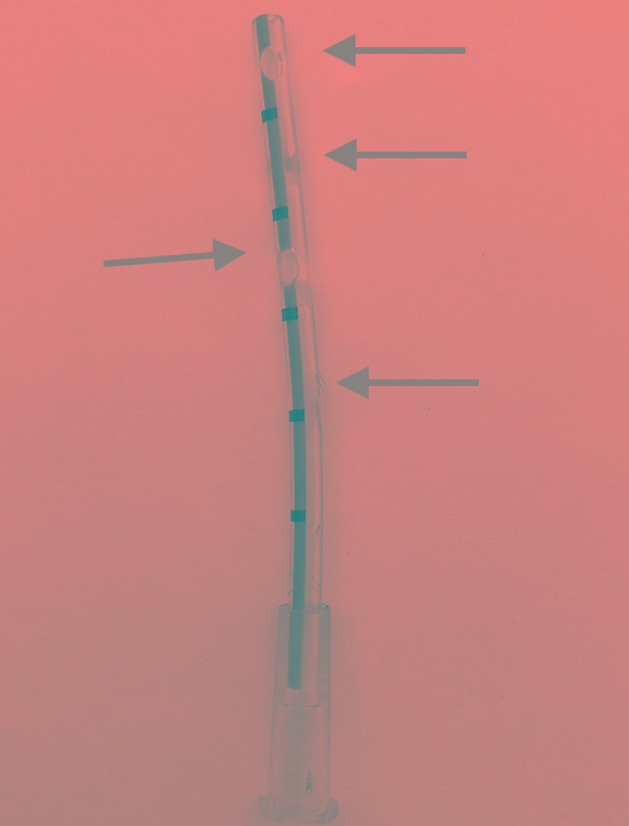
Modified intercostals drain (vygon 625.10)

The baby girl, weighing 960 gms, was born at 27 weeks of gestation to a mother with vaginal bleeding. She had respiratory distress syndrome and was commenced on nasal continuous positive airway pressure (CPAP) support. Umbilical lines were inserted and she was treated with intravenous (IV) fluids, parenteral nutrition, caffeine, and IV antibiotics. She was started on trophic feeds from day 2 of life. On day 8 of life, after receiving 5 mL 2 hourly expressed breast milk, she developed abdominal distension. The abdominal X-ray showed pneumoperitoneum (Fig. 1). The baby had developed necrotizing enterocolitis (NEC) stage IIIB. A peritoneal drain was inserted immediately. After 48 hours once the baby was stable, laparotomy was done. Multiple perforations were found in the cecum and ascending colon, so ileostomy was performed. The immediate post operative period was uneventful. Feeds were restarted postoperative day 4 (POD) (day 15). She reached full feeds on day 20. On day 39, on full feeds of 18 mL 2 hourly with full fortification, receiving calories of 130 Kcal/kg and proteins of 2.5 gm/kg, not receiving any nephrotoxic drugs, her urine output decreased. On day 40, she was anuric despite fluid boluses and had developed AKI. She became edematous and developed respiratory distress. Her investigations showed creatinine 1.8 mg/dL, urea 40 mg/dL, Na 125 mEq/L, and K 7.2 mEq/L. Her blood gas was suggestive of metabolic acidosis pH 7.2, pCO_2_ 28, pO_2_ 70, HCO_3_ 12.5, and base excess −12.5. A peripherally inserted central catheter (PICC) line was inserted and she was restarted on IV fluids, soda bicarbonate correction, potassium reducing measures, and furosemide infusion. She did not produce any urine for another 8 hours despite intensive measures. Her investigations: pH7.2, pCO_2_ 23, pO_2_ 82, HCO_3_ 11.7, BE-13.7, Na 122 mEq/L, K 6.8 mEq/L, and creatinine increased to 2.5 mg/dl. As per neonatal kidney disease improving global outcome (KDIGO) criteria AKI classification, she was in stage 2 of AKI with creatinine of 2.5 mg/dL and urine output <0.5 mL/kg/hour for 12 hours. It was decided to perform a peritoneal dialysis. Peritoneal dialysis was complicated to do in this baby because of the small size of abdomen and associated ileostomy. The standard available pediatric dialysis catheter was too long for her abdominal cavity. So, a 10 French intercostal drain (ICD) was chosen for the procedure. This has just one opening so three holes were created in it. The ICD was inserted in left para umbilical region. Small aliquots of 17 mL of 1.7% dialysate were used. Each cycle of 10 minutes in flow, 10 minutes dwell time, and 10 minutes outflow were started (Fig. 2). After 12 hours of doing dialysis, baby improved and started to make urine in the next 24 hours. Creatinine started to drop, serum electrolytes normalized, and urine output increased to 2 mL/kg/hour. Dialysis was stopped after 48 hours. The catheter was removed after 96 hours of insertion. There were no catheter related complications. Blood, urine and CSF culture grew *Candida albicans* for which fluconazole was started. She received 4 weeks of antifungal therapy. Baby was discharged to home with an ileostomy on day 78 at weight of 2 kg.

## DISCUSSION

Acute kidney injury is a known occurrence in sick neonates. Early recognition, identification of babies with risk factors, and monitoring of urine output and creatinine would help in recognizing the problem in time. There have been three large single center studies that have evaluated incidence of AKI in neonates weighing between 500 gm and 1500 gm. In these studies, the incidence of AKI varies from 18 to 43%.^1–3^ All the three studies have shown that AKI was independently associated with high mortality rate.

A recent multinational, multicenter, retrospective study AWAKEN has also shown a high incidence of 30% of AKI in sick neonates.^4^ It also reveals that preterm neonates with NEC stage II and III have 43% incidence of AKI. Infants with AKI have a four times higher independent odds of death and longer hospital length of stay than those without AKI.

Neonates especially ELBWs are susceptible to AKI as the developing glomeruli are subjected to rapid fluid shifts, nephrotoxic drugs and sepsis being a leading factor. Necrotizing enterocolitis and sepsis are leading causes of AKI in ELBWs.^5^ Our ELBW had both the risk factors.

The primary management for severe AKI is renal replacement therapy. Peritoneal dialysis is the mainstay of treatment when all conservative measures have failed. In term neonates, it is routinely performed and has shown good outcome if done in time. For term neonates, it can be easily done using the standard cuffed peritoneal dialysis catheter. It poses a challenge in preterm neonates especially ELBWs as dialysis catheters suitable for ELBW neonate are not available. Their small body size and inelastic abdominal wall makes the procedure challenging. The cuffed catheter length is 20 cm which is too long for small abdominal cavities of these tiny ELBWs.

We encountered a challenge regarding the choice of catheter to be used to do PD on our ELBW because the baby weighed just 1.2 kg and had an ileostomy. With such a small abdominal cavity, we anticipated problems such as leak of PD fluid around the ileostomy and the consequent risk of peritonitis due to the presence of ileostomy. We were also concerned of the fact that during the procedure, a bowel perforation may occur due to adherent bowel loops post NEC. In addition, the post NEC adhesions could complicate the insertion of catheter and also efficacy of the dialysis.

We used an intercostal drainage catheter (vygon ref 625.10 10 French Catheter), which is small and soft instead of the available peritoneal catheter, which is 20 cm long and too big for our baby.

There are several case reports of peritoneal dialysis being done in preterm neonates with AKI. Different innovative catheters have been used for PD. There are few case reports of PD in NEC with perforation. A primary peritoneal drain was put for perforation and peritoneal dialysis was carried out through the same. The survival rate of the babies was although poor. The authors concluded that the PD in NEC may also help to lavage out the inflammatory cytokines, toxins, and help in intestinal remodeling and healing.^6^

We could not find in the literature any similar case and are the first to report PD in an ELBW neonate with ileostomy post NEC.

## References

[1] Koralkar R,, Ambalavanan N,, Levitan EB,, McGwin G,, Goldstein S,, Askenazi D. (2011;). Acute kidney injury reduces survival in very low birth weight infants.. Pediatr Res..

[2] Viswanathan S,, Manyam B,, Azhibekov T,, Mhanna MJ. (2012;). Risk factors associated with acute kidney injury in extremely low birth weight (ELBW) infants.. Pediatr Nephrol..

[3] Carmody JB,, Swanson JR,, Rhone ET,, Charlton JR. (2014;). Recognition and reporting of AKI in very low birth weight infants.. Clin J Am Soc Nephrol..

[4] Jetton JG,, Boohaker LJ,, Sethi SK,, Wazir S,, Rohatgi S,, Soranno DE, (2017;). Incidence and outcomes of neonatal acute kidney injury (AWAKEN): a multicentre, multinational, observational cohort study.. Lancet Child Adolesc Health..

[5] Bakhoum CY,, Basalely A,, Koppel RI,, Sethna CB. Acute kidney injury in preterm infants with necrotizing enterocolitis.. J Matern Fetal Neonatal Med..

[6] Canpolat FE,, Yurdakök M,, Yiğit S,, Tekinalp G. (2010;). Can peritoneal dialysis be used in preterm infants with gastrointestinal perforation?. Pediatr Int..

